# Supplementary material for: Flat out Fabulous: How Barbie’s foot posture and occupations have changed over the decades, and the lessons we can learn
Source: PLoS One. 2025 May 14;20(5):e0323719. doi: 10.1371/journal.pone.0323719 (PMC12077710; doi:10.1371/journal.pone.0323719)
Supplement: Attachment — Submitted filename: Response to reviewers Feb 2025.docx [file pone.0323719.s003.docx]

1. **The introduction emphasizes Barbie's status as a social symbol, but does it need further explanation as to why "foot posture" was chosen as the core entry point of the study, rather than other physical or professional characteristics?**

The introduction has been modified to identify why foot posture is a measure of interest as it relates to Barbie’s symbolic status and the societal interest when her foot posture changed (page 4, lines 52-61)

Inserted text:

*The success of the 2023 Barbie movie (Universal Studios, Warner Bros Pictures) reinvigorated analysis on Barbie’s standing within social and cultural contexts. With a premise of “thanks to Barbie, all problems of feminism and equal rights have been solved” (1), ‘Barbieland’ is a seemingly perfect matriarchy. This is at least until “Stereotypical Barbie” (played by Margo Robbie) experiences an existential crisis presenting as flat feet. The equinus foot posture (where the ankle lacks adequate dorsiflexion or tip toe) is iconically associated with Barbie. So much so, that standing with feet flat on the floor was not only ‘gag-inducing’ in the movie, it launched the #Barbiefeetchallenge. This challenge asked participants to mimic the fixed equinus foot posture when exiting high-heeled shoes. While this seemingly celebrates the foot posture itself, it is a clear statement on Barbie’s preferred choice of high heeled footwear.*

No additional references added

2. **When introducing the impact of high-heeled shoes on health and the importance of changes in foot posture, the current research on the risk of lower limb injury caused by high-heeled shoes changing ankle joint function is unclear. "New insights optimize landing strategies to reduce the lower limb injury risk" (**[**https://doi.org/10.34133/cbsystems.0126**](https://url.au.m.mimecastprotect.com/s/sKx4Cvl1o9H7GkJ3YT8F5TQrgkT?domain=doi.org)**). The study highlights that ankle function is critical for lower limb stability and reducing the risk of injury during exercise. Studies have shown that changes in foot posture not only affect gait stability, but also reduce potential injury risk by optimizing impact force absorption. These findings provide a theoretical basis for understanding how different footwear choices adapt to functional requirements. Combined with the change in Barbie's foot posture from horseshoe to flat foot, this study provides scientific support for its biomechanical significance and further highlights the correlation between task demands and foot functional adjustment.**

Thank you for this reference however when we reviewed, it had limited discussion on shoes and their role in injury but instead ankle modelling when landing. We have reviewed and provide additional evidence supporting infrequent heel use, and ankle equinus during landing which identified risk of injury during exercise. With that said, we agree that clarifying that our statement around the impact of high heeled shoes on the lower limb as unclear relates across a broad spectrum of impact, such as foot morphology, skin and nail concerns, bony changes and/or pain/inflammation as well as injury – this is important given the purpose of our investigation was to identify the relationship between choice of heel height as it relates to need/task. Therefore, we have altered the introduction as below (page 4-5, lines 62-75):

Revised text:

*Outside of Barbie Land, aka ‘the real world’, health professionals have been discouraging the use of high heeled footwear since the early 1900’s (2, 3). Health professionals link high heel footwear as the cause of bunions (4, 5), knee osteoarthritis (6), plantar fasciitis (7, 8) and low back pain (9). Yet many of these health conditions are highly prevalent in the general population with low or no high heel use (10-12). It is also purported that excessive high heel wear results in ankle equinus and foot deformity, such as Barbie commonly displays. However, the majority of studies only investigate the immediate and short-term impact of high heeled shoes only (13), often in those that are non-wearers of high heels (14). Or there are studies where shoes with a heel pitch are atypically worn such as during high-intensity or sports related tasks (15, 16). From these investigations, we understand high heels have immediate and significant effects on walking gait; including reduced speed, shorter steps, and increased lower limb instability (17). But given there appears to be no known long-term studies on the use of high heeled shoes that mimic casual and considered use by participants, it begs the question if this evidence is relevant to the bulk of the relevant population?*

Additional references added to references already present:

6. Nguyen LY, Harris KD, Morelli KM, Tsai L-C. Increased knee flexion and varus moments during gait with high-heeled shoes: a systematic review and meta-analysis. Gait & Posture. 2021;85:117-25.

7. Wang M, Li S, Teo E-C, Fekete G, Gu Y. The influence of heel height on strain variation of plantar fascia during high heel shoes walking-combined musculoskeletal modeling and finite element analysis. Frontiers in Bioengineering and Biotechnology. 2021;9:791238.

15. Liu Z, Yamaguchi R, Fu S, Zhao H, Li Y, Kobayashi Y, et al. Epidemiology of ankle sprain and chronic ankle instability in elite adolescent dancesport athletes. The Physician and Sportsmedicine. 2024:1-10.

16. Xu D, Zhou H, Baker JS, István B, Gu Y. An investigation of differences in lower extremity biomechanics during single-leg landing from height using bionic shoes and normal shoes. Frontiers in Bioengineering and Biotechnology. 2021;9:679123.

3. **The authors used a custom FEET system to assess Barbie's foot posture and variety. Is the system proven? Can you provide evidence on the reliability and consistency of the classification criteria?**

The FEET classification system (Foot posture, Equity, Employment, and Time) was author developed, predominantly serendipitously as we realised the acronym fitted the manuscript theme so well. We did not formalise reliability/consistency of this system as outcomes were binary and simplistic (e.g., the options were flat or equinus for foot posture). We improved the robustness by having 10% (n = 275) of the dolls independently double assessed by three individual authors, concurrently with the first author independently completing all extraction. To clarify this, the manuscript has been altered as (page 7, line 175-180):

Revised text:

*As data extracted was binary (e.g., yes, no), we did not formally test reliability of this purpose-built and simple rating system. However, during its development, all authors met to clarify and refine the definitions and terminology for clarity. Then all data were extracted by a single author (CMW) into the custom developed spreadsheet, and 10% (n=275) of the data extracted were independently double checked by a second author (IG, KG or HB) to ensure correct categorisation against the FEET system.*

4. **Limited edition or special versions of Barbie (e.g. TV characters, limited collections) were excluded from this study. Does this bias the generality of the findings? What explains the scientific basis for these exclusion criteria?**

The premise of this study was that Barbie may reflect footwear choices that high heeled shoe wearers, predominantly a female population, would make on a daily basis. The limited edition dolls are, by nature, outliers and potentially not meet this criteria due to their development for limited cultural or advertising purposes.

There was also a pragmatic rationale that these dolls are harder to source and harder to obtain accurate pictures of their foot position. Their introduction would have potentially introduced error into the FEET system. We could not guarantee inclusion of all limited edition dolls due to potentially limited inclusion or information in online catalogues. We have added the words “to ensure outcomes related to the ‘mass market’, we excluded any Barbie or friend that had limited reach…” to the manuscript for clarity (page 6, line 157-158).

Inserted text:

*To ensure outcomes related to the ‘mass market we excluded any Barbie or friend that had limited reach…*

5. **Data mainly comes from online catalogs and personal collections. Is it possible that there are systematic biases in the accuracy and completeness of this information? Is the impact of differences in these sources on the results considered?**

We believe that the use of multiple sources to cross check information strengthens the final list however see that we did not appropriate describe how we used the catalogues as a method of data collection robustness. We used personal collections as a method to develop the images. This information has been revised (page 6, Lines 149-151).

Added:

*We used multiple online catalogues as a method to cross check the accuracy and completeness of Barbie’s names across the years. We used the images in the sales sites and personal collections to cross check accuracy of foot postures and develop images as examples for the FEET system.*

We have also already listed in the limitations of this method at page page 10, line 258-9.

6. **The results show that Barbie's flat foot posture is highly correlated with occupation, but these correlations are based on statistical analysis only. Can more care be taken to avoid interpreting statistical correlations directly as causation?**

We have taken care within the results to only report correlation and frequencies at each time point as succinctly as possible without inference of causation. We have removed the p values from the results and added the 95% CIs into the results and figure, to minimise misinterpretation of the results.

We have also changed the term relationship to correlation in the discussion at page 9, line 250

7. **Although the charts (such as Figure 3 and Figure 4) show time and correlation trends, can more specific data annotations (such as standard errors or confidence intervals) be provided in the charts to support the confidence of the results?**

95%CI added within Figure 4.

8**. Barbie's sample size varies widely across time periods (e.g., there were only 330 Barbie samples in 2020-2024 compared to 879 in 2000-2009). Could this uneven distribution of samples affect the results of statistical analysis?**

While it is possible, it is unlikely. The following has been added, with reference to sample size for correlation into the limitations at page 10 lines 257-8:

Added:

*The sample size, even with variation across the time period is robust, allowing for stable estimates (36)*.

Reference

3.6 Schönbrodt FD, Perugini M. At what sample size do correlations stabilize? Journal of Research in Personality. 2013;47(5):609-12.

**9. The study notes that "changes in Barbie's foot posture may reflect dynamic changes in social employment patterns and policies." Can this inference be further explored in a broader sociological or culturological context?**

Added historical context at page 9, 241-249:

*This change in employment representation of Barbie mimics the change in women’s employment across the world (30). This increase in numbers of women employed, inevitably also impacts the differing roles that women play in the workforce. Aligning with these changes is the increase in employment equity enshrined in discrimination laws, particularly in the USA which Barbie originates. The frequency of Barbie in employment roles rapidly increase from the 1980’s, potentially also aligning with a landmark change in USA laws where women could independently own business without a male relatives permission (H.R.5050 - Women's Business Ownership Act of 1988) (31). Society changing together with expectations of role and laws enabling these changes, means a doll that represents women, also changes in how looks and dresses.*

References:

30.	Our world in data. Female participation in labor markets grew remarkably in the 20th century: 2024; 2023 [Available from: <https://ourworldindata.org/female-labor-supply#:~:text=all%20female%20workers.-,Female%20labor%20force%20participation%20worldwide%20is%20higher%20today%20than%20several,of%20countries%2C%20across%20income%20levels>.

31.	Women's Business Ownership Act of 1988, USA Congress(1987-1988).

**10. The discussion section suggests that "health professionals should reduce negative perceptions of high heels." Is there a lack of direct evidence to support this suggestion? Does more literature or further analysis of Barbie data need to be done to support this?**

We have increased the references to health professionals bias of footwear in the introduction in relation to point 2. We have also added a referenced statement to the discussion at page 9, lines 260-261, pointing to popular media and their statements of the dangers of high heel wearing.

Added:

*This may be a more appropriate approach to guiding footwear advice, than castigating high heel wearing often seen in medical and journalistic websites (34, 35).*

References:

34. WebMD. Worst shoes for your feet 2023 [Available from: https://www.webmd.com/pain-management/ss/slideshow-worst-shoes-for-your-feet.

35.Heoepfner F. Gen Z loves Stilettos. So do Women over 50. The Wall Street Journal, 2024.

Reviewer 2:

**I have read through this paper several times and spent a fair amount of time reflecting on the meaning of this paper as well as the potential contribution it can make to the literature. In my opinion, the work has no scientific value and falls within the realm of fantasy and speculation. It adds nothing from a clinical point of view. Therefore, I do not recommend the work for further proceedings.**

We must counter Reviewer 2’s critique of our paper. Our counter is that by definition research, is about generating new knowledge. Our approach was systematic, with a clear aim, definitions, and variables reported in a way that other research teams can replicate and therefore generated new knowledge.

We also wish to clear up some misconceptions: Fantasy is imagining the impossible (Oxford dictionary definition), and speculation is forming a theory without evidence (Oxford dictionary definition). Neither term applies to our research as we have presented it.

Our research team consists of clinicians who discuss footwear with people daily. The Barbie movie sparked global interest in ankle equinus and high heel wearing, and our discussions on this topic were both clinical and social. This research idea was generated directly from our clinical work.

We acknowledge that this paper might not sway those who believe all high heels are inherently harmful to foot health. However, we hope it opens the door to evidence-based conversations about foot health and footwear choices, supporting autonomy of adults to make footwear choices that align with their lifestyles.

We have not made any changes to the article based on Reviewer 2’s statement, as there was no suggestions for changes. We leave the decision for action in the hands of the editor.
